# Health literacy, awareness and referral patterns of mothers in childhood vaccination: a cross-sectional study in Iran

**DOI:** 10.1186/s12889-025-25905-0

**Published:** 2025-12-23

**Authors:** Elaheh lael-Monfared, Samaneh Sabouri, Negin Rahmani, Nooshin Peyman

**Affiliations:** 1 Department of Health Education and Promotion, School of Public Health, Mashhad University of Medical Sciences, Mashhad, Iran; 2 Social Determinants of Health Research Center, Mashhad University of Medical Sciences, Mashhad, Iran; 3 Department of Biostatistics, Faculty of Health, Mashhad University of Medical Sciences, Mashhad, Iran; 4 Student research committee, Mashhad University of medical sciences, Mashhad, Iran

**Keywords:** Vaccination, Awareness, Health literacy, Children, Referral patterns

## Abstract

**Introduction:**

Vaccination of children is one of the most effective strategies for preventing preventable infectious diseases in childhood. Additionally, successful control of these diseases requires timely vaccination. This study aimed to determine maternal health literacy, awareness of the importance of timely immunization and the referral patterns of Iranian mothers for the immunization of their children.

**Methods:**

This cross-sectional study was conducted among 250 mothers attending health centers using a multi-stage cluster sampling approach. Health centers were considered as clusters, from which two clusters and subsequently two comprehensive health service centers within each cluster were randomly selected. Eligible mothers attending these centers were then recruited. Data were collected using validated questionnaires, including demographic information, health literacy (s-TOFHLA), and awareness of the importance of timely vaccination. After confirming the validity and reliability of the tools, the data were analyzed with SPSS version 22. Descriptive statistics were applied alongside Chi-square, Kruskal-Wallis, and correlation tests to examine associations.

**Results:**

Mothers’ awareness of timely vaccination was moderate (12.73 ± 2.04), and the mean health literacy score was 85.2 ± 11.8. Only 40.8% reported regular vaccination visits. Awareness significantly increased with maternal age (highest rank mean in > 30 years: 135.6, *p* < 0.001) and education (highest in bachelor’s degree or higher: 138.9, *p* = 0.002), while employed mothers showed the highest awareness levels compared with housewives and self-employed mothers (rank mean 147.7, *p* < 0.001). Birth order was not significantly related to awareness (*p* = 0.769). Regular vaccination visits were more common among mothers who received information from physicians or health centers (72.5%, *p* = 0.011) and among mothers older than 30 years (49%, *p* = 0.046). Although mothers with adequate health literacy and first-born children had higher rates of regular visits, these associations were not statistically significant.

**Conclusion:**

Maternal age, education, occupation, and information sources play an important role in vaccination awareness and adherence. Targeted educational and community-based interventions to improve maternal health literacy and awareness may enhance timely vaccination and reduce the impact of misinformation.

## What is Already Known on This Topic?

- Vaccination is a key strategy for preventing infectious diseases in children, and timely immunization is crucial for effective disease control.
- Maternal health literacy significantly impacts children’s vaccination rates and overall health outcomes.
- Awareness of the importance of vaccinations varies among mothers, influenced by factors such as education, occupation, and access to information.

## What This Study Adds

- The study provides quantitative insights into Iranian mothers’ awareness of the importance of timely vaccination, revealing a moderate level of awareness.
- It highlights the correlation between maternal demographics (age, occupation, education) and their awareness and behaviors related to vaccination.
- The study identifies the need for improved health literacy and targeted interventions to enhance mothers’ understanding of vaccination importance.

## How This Study Might Affect Research, Practice, or Policy

- The findings can guide future research on maternal health literacy and its direct impact on vaccination rates, especially in different cultural contexts.
- Health policymakers can use this data to develop targeted educational programs aimed at increasing awareness and promoting regular vaccination visits among mothers.
- The study underscores the necessity for healthcare providers to engage with mothers through effective communication strategies, ensuring they receive accurate information about vaccinations.

## Introduction

Vaccines play a fundamental role in public health and are one of the most significant achievements of medical science, preventing and controlling dangerous diseases [1]. They are among the most cost-effective interventions, substantially reducing morbidity, mortality, and healthcare costs, particularly among children [2, 3]. Childhood vaccinations have led to remarkable reductions in mortality, exemplified by the eradication of smallpox [4, 5]. Despite these successes, recent years have witnessed a decline in coverage levels across several countries, with rates still falling short of the global target of 90% [3].

Successful control of vaccine-preventable diseases depends not only on achieving high coverage but also on the timely administration of vaccines [6]. However, vaccine hesitancy—defined as delay or refusal despite availability—remains a major challenge worldwide [7]. Studies from Armenia, China, Australia, and Singapore show that many children fail to receive recommended vaccines on time [8, 9]. Delayed or incomplete vaccination has been linked to parental dissatisfaction, concerns about vaccine safety and efficacy, and misinformation [10]. While many parents acknowledge the protective role of vaccines, knowledge gaps and misconceptions remain common [11].

Global data highlight persistent gaps in vaccination coverage. In 2023, 14.5 million children were categorized as zero-dose, having received no vaccines. Coverage of the third dose of diphtheria, tetanus, and pertussis vaccine (DTP3) was 84%, and measles first-dose coverage was 83%—both below the 2019 levels [16]. Coverage varies substantially across regions and countries, with some falling short of herd immunity thresholds [3, 12, 13].

In Iran, overall vaccination coverage exceeds 98%, but this still leaves around 11,000 children unvaccinated. Moreover, delayed vaccination remains a significant issue, with prevalence ranging from 42% to over 90% depending on the region [14, 15]. Given the mobility between Iran and neighboring countries with weaker vaccination systems, maintaining timely and complete vaccination is a critical public health priority [16].

Parental health literacy plays an essential role in shaping health behaviors, including vaccination. Health literacy is defined as the ability to obtain, process, and understand health information in order to make informed decisions [17, 18]. Higher health literacy has been associated with greater parental involvement and more positive attitudes toward vaccination [19]. Conversely, low health literacy contributes to vaccine hesitancy and poorer adherence to recommended schedules [20]. As parents are the primary decision-makers for child health [21], lack of awareness about the importance of timely vaccination may contribute to delays or refusals [10, 14]. Based on existing evidence, we hypothesized that higher maternal health literacy and education would be associated with greater awareness of vaccination importance and more regular attendance at vaccination appointments. Therefore, this study aimed to investigate maternal health literacy, awareness of the importance of vaccination, and referral patterns for vaccination among Iranian mothers.

## Materials and methods

### Study design and sample size

This cross-sectional analytical study was conducted from May to July 2024 to examine the relationship between general health literacy and vaccination knowledge among mothers of children under 7 years old attending health centers in Mashhad, Iran. All participants were informed about the study objectives, confidentiality of data, and research procedures before providing written informed consent and completing the questionnaires. A multi-stage cluster sampling method was employed. Each health center was considered a cluster. Among the five health centers in Mashhad, one cluster was randomly selected, and then four comprehensive health service centers under that health center were randomly chosen. Mothers visiting these centers were approached consecutively. A total of 250 mothers were invited, and all agreed to participate in the study; no participants were excluded.

The required sample size was calculated using G*Power software, assuming a correlation coefficient of 0.3, a type I error of 5%, and a type II error of 10% (90% power). The minimum required sample size was 238, and with an anticipated 10% attrition rate, the final sample size was set at 250 participants.

## Data collection tools

*Short Form of the Test of Functional Health Literacy in Adults (TOFHLA-S)*: This 40-item tool includes two sections: Reading Comprehension (36 items): Measures the ability to understand health-related texts such as imaging instructions and patient rights documents (score: 0–72). Numeracy (4 items): Assesses the ability to perform simple calculations in health contexts, such as interpreting prescriptions and test results (score: 0–28). The total score ranges from 0 to 100 and is classified as inadequate (0–59), borderline (60–74), and adequate health literacy (75–100). The Persian version has confirmed validity and reliability, with Cronbach’s alpha values of 0.69 for numeracy and 0.78 for reading comprehension [22, 23].

*Vaccination knowledge questionnaire*: This 18-item questionnaire evaluates maternal knowledge based on the National Vaccination Program and guidebook. Each item is scored as “yes” or “no,” with one point for each correct answer. The total score ranges from 0 to 18, classified as poor (0–9), moderate (10–15), or good (16–18). Its validity and reliability were confirmed in a study by Pourali et al., with a reliability coefficient of 0.79 [24]. The questionnaires were primarily self-administered, and the average time to complete them was approximately 10–15 min. For mothers who had difficulties (e.g., low literacy), the researcher assisted in an interviewer-led manner.

Vaccination visit Pattern was initially collected through mothers’ self-reports. To ensure accuracy, these reports were cross-checked with the children’s vaccination cards during the data collection process.

### Statistical analysis

Data were analyzed using SPSS version 22. Descriptive statistics, including frequencies, percentages, means, and standard deviations, were calculated to summarize participants’ demographic characteristics, maternal vaccination knowledge, health literacy, and vaccination attendance patterns. The normality of continuous variables was assessed using the Shapiro–Wilk test, which indicated that the data were not normally distributed. Therefore, non-parametric tests were applied. Specifically, the Kruskal–Wallis test was used to compare maternal vaccination knowledge scores across demographic subgroups, vaccination visit patterns, and health literacy levels. For pairwise comparisons between groups, a post-hoc Dunn’s test with Bonferroni correction was applied. In addition, eta-squared (η²) was reported as the measure of effect size for the Kruskal-Wallis test [25]. Its value ranges from 0 to 1, representing the proportion of total variance in the ranked data that is attributable to the differences between groups. Maternal health literacy was categorized into three groups: inadequate, borderline, and adequate, while maternal knowledge was categorized into three levels: poor, moderate, and good. The relationship between the categorical variables was analyzed using a chi-square test. The strength of the association was measured by Cramer’s V, a statistic that ranges from 0 to 1, with higher values indicating a stronger association. A p-value of < 0.05 was considered statistically significant.

## Results

In this study, 250 mothers attending comprehensive health service centers in Mashhad were surveyed. The majority of participants (59.8%) were over 30 years old, and more than half (53.4%) were housewives. Regarding education, 43.8% held a bachelor’s degree or higher. Firstborn children accounted for 43.4% of cases. Physicians and health centers were cited as the primary and top-priority source of vaccination information by 58.6% of mothers, and 42.9% reported visiting health centers regularly. (Table 1).

**Table 1:** Frequency distribution of participants’ demographic characteristics (*n* = 251)

Variable	Categories	*N*	(%)
Mother’s Age	Less than 20 years	10	4.0%
	20–25 years	35	13.9%
	25–30 years	55	21.9%
	More than 30 years	150	59.8%
Mother’s Occupation	Employed	84	33.5%
	Housewife	134	53.4%
	Self-employed	32	12.7%
Mother’s Education	Below high school diploma	30	12.0%
	High school diploma	68	27.1%
	Associate’s degree	42	16.7%
	Bachelor’s degree or higher	110	43.8%
Child’s Birth Order	First child	109	43.4%
	Second child	84	33.5%
	Third child	38	15.1%
	Fourth or higher	19	7.6%
Source of Information	Mass media	72	28.7%
	Books and newspapers	15	6.0%
	Physicians and health centers	147	58.6%
	Relatives and friends	16	6.4%

The mean maternal vaccination knowledge score was 12.73 ± 2.04 (range 3–17), with 88.4% having moderate knowledge and 6.4% demonstrating good knowledge. The mean health literacy score was 85.2 ± 11.8, indicating an overall adequate level. Variability in awareness was observed across the 18 knowledge items (Table 2).

**Table 2:** Frequency distribution of responses to maternal awareness questions about the National vaccination program

Questions	No	(%)	Yes	(%)
1. Should a child receive a vaccine before starting school?	8	3.2%	242	96.8%
2. Should the national vaccination program start from birth?	6	2.4%	244	97.6%
3. Should a vaccine be administered to adolescents in high school?	30	12%	220	88%
4. If high fever occurs after vaccination, should acetaminophen be used for the next dose?	39	15.6%	211	84.4%
5. Is vaccination an effective and appropriate method for preventing infectious diseases?	13	5.2%	237	94.8%
6. Is it important to attend vaccination on the date written on the vaccination card?	12	4.8%	238	95.2%
7. Can the national vaccination program protect children from serious related diseases?	9	3.6%	241	96.4%
8. Should vaccination be postponed due to fever?	44	17.6%	206	82.4%
9. Is the hepatitis B vaccine included in the national vaccination program?	100	40%	150	60%
10. Is vaccination prohibited in children with a cold?	41	16.4%	209	83.6%
11. If an apparently healthy child is using antibiotics, should vaccination be postponed?	70	28%	180	72%
12. Is vaccination prohibited in children with diarrhea?	58	23.2%	192	76.8%
13. Does vaccination against pertussis provide 100% protection?	137	54.8%	113	45.2%
14. Does the national vaccination program usually cause serious complications in children?	208	83.2%	42	16.8%
15. Does vaccination weaken a child’s immune system due to disease prevention?	206	82.4%	44	17.6%
16. If parents do not bring the child for vaccination on time, should vaccination be discontinued?	241	96.4%	9	3.6%
17. Should breastfeeding be stopped when giving oral polio drops?	188	75.2%	62	24.8%
18. Is fever a sign of vaccine injection?	225	90%	25	10%

 The correct answer is gray houses**.**


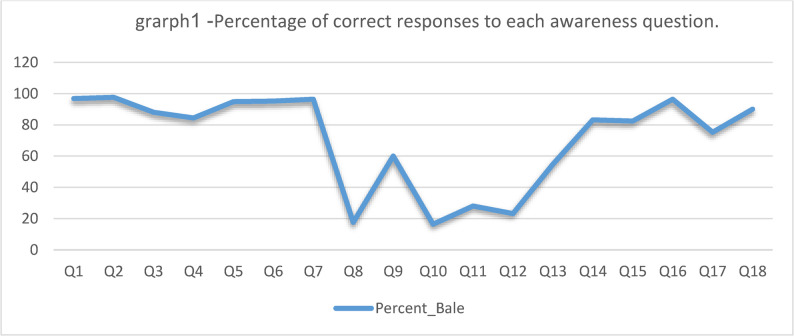


The analysis of line graph 1 related to 18 vaccination awareness questions showed that participants were highly aware of some questions, while the correct answer rate was significantly lower for others.

Kruskal-Wallis tests revealed significant differences in maternal knowledge scores based on demographic characteristics. Older mothers (> 30 years) had higher knowledge scores (*p* < 0.001), employed mothers scored higher than housewives or self-employed mothers (*p* = 0.001), and mothers with a bachelor’s degree or higher had the highest scores (*p* = 0.002). However, no significant associations were found between child birth order, source of information, or vaccination visit pattern and maternal knowledge (*p* > 0.05), suggesting that vaccination adherence is a multifactorial phenomenon and not solely determined by maternal knowledge (Table 3(.

**Table 3:** Comparison of awareness scores based on demographic variables, vaccination visit pattern, and health literacy level

Variable	Groups	*N*	Median	IQR	Test Statistic	*p*-value	$\:{\boldsymbol{\upeta\:}}^{2}$
Mother’s Age	Less than 20 years^a^	10	10	2.5	18.498	< 0.001	0.738
	20–25 years	35	12	2			
	25–30 years^b^	55	13	3			
	More than 30 years^b^	150	13	2			
Mother’s Occupation	Housewife^b^	134	13	3	15.155	< 0.001	0.053
	Employed^a^	84	13	3			
	Self-employed^b^	32	12	2			
Child’s Birth Order	First child	109	13	3	1.134	0.769	0.008
	Second child	84	13	2			
	Third child	38	12	3			
	Fourth or higher	19	12	3			
Mother’s Education	Below high school diploma^b^	30	12	2.5	14.414	0.002	0.046
	High school diploma^b^	68	12	3			
	Associate’s degree	42	13	2			
	Bachelor’s degree or higher^a^	110	13	2			
Source of Information	Mass media	72	13	3	3.626	0.305	0.046
	Books and newspapers	15	13	3			
	Physician/Health center	147	13	2			
	Relatives and friends	16	13	2			
Vaccination visit Pattern	Regular	102	13	2	1.289	0.525	0.003
	Irregular	31	12	3			
	Only when needed	105	13	2.5			
Health Literacy	Inadequate	10	13.5	3	0.913	0.634	0.004
	Borderline	24	12.5	3			
	Adequate	216	13	2.75			

Significance values have been adjusted by the Bonferroni correction for multiple tests. a, b: Different letters indicate a statistically significant difference between groups (*p* < 0.05) based on a post-hoc Dunn’s test with Bonferroni correction

Regarding vaccination attendance, only 40.8% of mothers visited health centers regularly for their children’s vaccinations. In contrast, 12.4% visited irregularly, and 46.8% visited only when necessary. Regular attendance was highest among mothers who reported physicians or health centers as their main source of vaccination information (72.5%). Housewives and mothers of firstborn children also had relatively higher rates of regular visits (51%), although these differences were not statistically significant.

Mothers with a bachelor’s degree or higher had the highest regular visit rate (47.1%), while age was significantly associated with regular visits (*p* = 0.046), with mothers over 30 years more likely to visit regularly (49%). Although mothers with adequate health literacy had the highest proportion of regular visits (83.3%), this association did not reach statistical significance (*p* = 0.496). This lack of statistical significance may be due to the relatively small sample size, potential confounding factors, or other sociodemographic and behavioral variables influencing vaccination adherence. Additionally, no significant relationship was found between maternal knowledge level and vaccination visit pattern (*p* = 0.692). These results indicate that while health literacy and knowledge are important, vaccination adherence is influenced by multiple factors, including source of information and maternal age (Table 4).

**Table 4:** Frequency distribution of vaccination attendance pattern based on demographic variables, awareness, and health literacy

Variable		Regular Visit *N* (%)	Irregular visit *N* (%)	Only When Needed *N* (%)	χ²	*p*-value	Cramer’s V
Mother’s Age	Less than 20 years	7 (6.9)	2 (6.5)	1 (0.9)	12.83	0.046	0.16
	20–25 years	18 (17.6)	2 (6.5)	15 (12.8)			
	25–30 years	27 (26.5)	6 (19.4)	22 (18.8)			
	More than 30 years	50 (49.0)	21 (67.7)	79 (67.5)			
Mother’s Occupation	Housewife	35 (34.3)	10 (32.3)	39 (33.3)	0.896	0.925	0.042
	Employed	52 (51.0)	18 (58.1)	64 (54.7)			
	Self-employed	15 (14.7)	3 (9.7)	14 (12.0)			
Child’s Birth Order	First child	52 (51.0)	12 (38.7)	45 (38.5)	6.626	0.357	0.115
	Second child	32 (31.4)	13 (41.9)	39 (33.3)			
	Third child	11 (10.8)	5 (16.1)	22 (18.8)			
	Fourth or higher	7 (6.9)	1 (3.2)	11 (9.4)			
Mother’s Education	Below high school diploma	15 (14.7)	5 (16.1)	10 (8.5)	4.557	0.602	0.095
	High school diploma	23 (22.5)	9 (29.0)	36 (30.8)			
	Associate’s degree	16 (15.7)	6 (19.4)	20 (17.1)			
	Bachelor’s degree or higher	48 (47.1)	11 (35.5)	51 (43.6)			
Source of Information	Mass media	18 (17.6)	9 (29.0)	45 (38.5)	16.588	0.011	0.182
	Books and newspapers	6 (5.9)	1 (3.2)	8 (6.8)			
	Physician/Health center	74 (72.5)	18 (58.1)	55 (47.0)			
	Relatives and friends	4 (3.9)	3 (9.7)	9 (7.7)			
Health Literacy	Inadequate	6 (5.9)	0 (0.0)	4 (3.4)	3.384	0.496	0.082
	Borderline	11 (10.8)	4 (12.9)	9 (7.7)			
	Adequate	85 (83.3)	27 (87.1)	104 (88.9)			
Awareness	Weak	5 (4.9)	1 (3.2)	7 (6.0)	2.240	0.692	0.067
	Average	88 (86.3)	28 (90.3)	105 (89.7)			
	Good	9 (8.8)	2 (6.5)	5 (4.3)			

Table 5 presents the results of the multivariate logistic regression analysis examining factors associated with vaccination referral patterns. After adjusting for potential confounding variables, maternal age and source of information were identified as significant predictors of referral behavior, whereas education level, occupation, and health literacy were not independently associated.

**Table 5:** Multivariate logistic regression analysis of factors associated with vaccination referral patterns

Variable		B	SE	OR	*p*-value
Mother’s Age	Less than 20 years	2.141	0.832	8.505	0.005
	20–25 years	1.097	0.453	2.997	
	25–30 years	0.892	0.365	2.441	
	More than 30 years	-	-	-	
Mother’s Occupation	Housewife	− 0.840	0.463	0.432	0.136
	Employed	− 0.954	0.516	0.385	
	Self-employed	-	-	-	
Child’s Birth Order	First child	0.377	0.605	1.458	0.244
	Second child	− 0.219	0.605	0.804	
	Third child	− 0.377	0.665	0.686	
	Fourth or higher	-	-	-	
Mother’s Education	Below high school diploma	− 0.054	0.561	0.947	0.829
	High school diploma	− 0.342	0.431	0.711	
	Associate’s degree	− 0.292	0.454	0.747	
	Bachelor’s degree or higher	-	-	-	
Source of Information	Mass media	− 0.205	0.703	0.814	0.000
	Books and newspapers	1.155	0.864	3.175	
	Physician/Health center	1.349	0.665	3.852	
	Relatives and friends	-	-	-	
Health Literacy	Inadequate	0.804	0.741	2.234	0.183
	Borderline	0.814	0.515	2.256	
	Adequate	-	-	-	
Awareness	Weak	−2.120	0.913	0.120	0.059
	Average	−1.096	0.588	0.334	
	Good	-	-	-	

Reference category for each variable is indicated by “–”. *B* Regression coefficient, *SE* Standard Error, *OR* Odds Ratio; *p* < 0.05 considered statistically significant

## Discussion

Vaccination schedules and the administration of each vaccine are determined by the point at which maternal antibodies have declined and the immune system is ready to respond to the vaccine components. This timing is crucial because it coincides with the age at which the disease has the greatest impact on morbidity and mortality [6]. Delays in administering vaccines can prolong susceptibility to diseases, potentially leading to serious health consequences and reduced vaccine efficacy [26].

The results of this study indicate that parental awareness of the importance of vaccinating children is at a moderate level. In a study conducted by Pourali et al., approximately 75.8% of mother’s demonstrated moderate awareness, about 15% had poor awareness, and around 8% exhibited good awareness [26]. A systematic review published in 2024 highlighted significant variability in parental awareness of child vaccination, with 52 studies reporting levels ranging from 6.5% to 100%. Parental awareness tends to be relatively high in European and North American countries, whereas it is lower and more inconsistent in some African and Asian countries [27]. Zagminas et al. reported that about one-fifth of parents lack sufficient information regarding vaccinations [28]. Similar findings have been observed in studies conducted in Lebanon and other Arab countries [29, 30]. Furthermore, studies from Yemen and Malaysia have reported parental knowledge levels ranging from low to moderate [31, 32].

This study indicates that while over 95% of mothers acknowledge the importance of timely and regular vaccination visits, less than half actually take the necessary steps to ensure their children are vaccinated on schedule. In one research study, it was found that 95.5% of mothers considered vaccination essential; however, more than half of the children did not receive their vaccines at the appropriate times, and nearly half of the mothers were unaware of the names of the vaccines or the diseases they prevent [33]. This disparity between attitudes and actual behaviors may be influenced by several factors. Barriers such as difficulties in accessing health centers, transportation issues, family responsibilities, and a lack of social support may prevent mothers from visiting on time. Additionally, misinformation and concerns about vaccine safety, particularly from social media sources, can affect decision-making, even when parents recognize the importance of vaccines [16, 34, 35].

Although health professionals remain the primary source of reliable vaccination information, the internet and social media are commonly used for health-related inquiries, which can expose parents to both accurate and misleading content [36]. Studies indicate that exposure to vaccine-critical websites or blogs can negatively influence attitudes toward vaccination [37, 38]. To address this, educational interventions should provide accurate information, integrate culturally sensitive communication, and include strategies to correct misconceptions and foster positive emotional engagement.

The current study shows that demographic factors like age, education, and employment significantly affect mothers’ vaccination awareness. Mothers over 30, with university education, and formal employment tend to have higher awareness. Age and information sources also influence regular vaccination visits. Studies from Iran and globally identify factors such as low maternal education, poor healthcare communication, employment status, and perception that vaccination is unnecessary as reasons for irregular visits [6, 8, 9, 14]. Older maternal age and lower birth order are linked to higher vaccination completion. Quality of interaction with healthcare providers also affects vaccination regularity [39, 40]. Similar findings appear in Nigeria and Ghana, where older, educated mothers show greater awareness and children are more likely vaccinated [41, 42]. However, other studies highlight roles of occupation, prenatal care, delivery place, and access to health centers [43]. These differences show socioeconomic and local health system factors influence vaccination behavior even within the same country. Some multivariate models suggest age and education are more critical than occupation for awareness [44]. Additionally, lack of awareness, information sources, health literacy, poor advice, and limited understanding of vaccination benefits and risks also shape parental vaccination behavior [45–48]. Overall, these findings reveal a complex interplay of factors affecting timely childhood vaccinations.

According to the results of this study, there is no significant relationship between health literacy and the awareness of the importance of vaccination or referral for vaccination. The impact of health literacy on health outcomes varies across different social groups [7]. Research on maternal health literacy and childhood vaccination yields mixed results. Some studies have found a positive association between maternal health literacy and vaccination rates [20], while others report no significant relationship [49]. Additionally, Pati et al. found no association between health literacy and vaccination adherence [50]. However, the role of maternal health literacy in predicting immunization status remains unclear. Examining the role of health literacy could provide insight into how other factors influence immunization status. For example, having adequate health literacy may lessen the effects of various sociodemographic characteristics on immunization status.

In this study, child birth order, source of information, and patterns of health center visits were not significantly associated with mothers’ knowledge scores. A systematic review indicated that vaccination adherence is more strongly influenced by a set of interrelated factors—such as trust in the health system, social norms, and perceptions of risk—rather than by single variables like birth order or source of information [51]. It appears that these variables, when considered in isolation, may have only a limited effect, and their true impact is likely shaped by broader socio-psychological factors such as maternal health literacy, educational level, access to services, and cultural attitudes. The lack of significant associations observed in this study may also be explained by the relatively uniform access of most mothers to formal information sources (e.g., physicians and health centers), the limited sample size, or the influence of confounding factors. Moreover, adherence to vaccination visits is influenced by a wide range of social, economic, and cultural determinants, and maternal knowledge alone may not be sufficient to predict such behaviors. This is consistent with findings from other studies that have described vaccination adherence as a multifactorial phenomenon. Ultimately, these results highlight the need for educational programs and promotional interventions to go beyond knowledge enhancement, addressing practical, motivational, and accessibility aspects of maternal engagement with vaccination services.

In this multivariate analysis, maternal age and source of information emerged as the strongest independent predictors of vaccination referral patterns. Mothers younger than 20 years had markedly higher odds of reporting complete and timely vaccination referrals compared with mothers over 30 (OR = 8.50). Although age effects on vaccine behavior are inconsistent across studies, some recent descriptive studies have reported important differences in uptake by maternal age, which may reflect differential access to services, parity, or health-seeking behavior across age groups [52]. The observed strong association between professional sources of information and appropriate referral behavior supports the well-established role of healthcare providers in promoting vaccination. A health-professional recommendation has repeatedly been shown to be one of the most influential determinants of vaccine uptake across settings and vaccines. Our finding that information from physicians/health centers (OR ≈ 3.85) was associated with substantially higher odds of timely referral aligns with prior work demonstrating the impact of provider recommendation on parental vaccination decisions [53].

Information obtained via books and newspapers was also associated with higher odds of appropriate referral (OR ≈ 3.17). This suggests that, in our study population, traditional media and printed educational materials may play a positive role in raising awareness and facilitating appropriate vaccination behavior — a pattern observed in other recent investigations of media and information sources. However, the influence of media is complex and may be mediated by the content quality and by pre-existing attitudes toward vaccination [54]. Interestingly, neither health literacy nor awareness reached statistical significance in the adjusted model, despite point estimates suggesting better referral behavior with higher literacy and awareness. This may reflect limited statistical power, residual confounding, or the possibility that the effects of literacy and awareness operate indirectly through interactions with information sources and provider contact. Systematic reviews have reported inconsistent but generally positive associations between health literacy (or vaccine literacy) and vaccine intentions/status, indicating that the relationship can vary by context and by how literacy is measured [55].

When interpreting the results, several limitations of this study should be acknowledged. As a cross-sectional design, the study restricts the ability to draw causal inferences. Additionally, there may be underlying factors or specific health-related dimensions, influencing the relationships between health literacy, awareness of vaccination importance, and adherence to regular medical visits. Therefore, future research is recommended to examine additional variables, particularly the impact of cyberspace, family support, and self-efficacy in overcoming barriers. Furthermore, exploring other dimensions of health literacy, including media health literacy and e-health literacy among mothers, is warranted. Health centers, in collaboration with social networks, should develop effective information campaigns targeting parents to counter misinformation and enhance adherence to childhood vaccination schedules. Another limitation of this study is that no statistical correction (e.g., Bonferroni correction) was applied for multiple testing. However, since the primary aim of the study was to identify potential associations rather than test definitive hypotheses, the possibility of type I error should be considered when interpreting the results. In this study, only the knowledge and practice (referral behavior) dimensions of parental vaccination were assessed, and the attitude component was not measured due to the design of the study and the focus of the questionnaires. Therefore, the findings should be interpreted with this conceptual limitation in mind. Future studies may consider including the attitude dimension to provide a more comprehensive evaluation of parental influences on vaccination behavior.

Future studies could expand on the findings of this research by exploring additional factors that influence childhood vaccination. Specifically, investigating vaccine hesitancy among mothers, including its social, cultural, psychological, and informational determinants, may offer further insights into vaccination behavior. It is also recommended that future research examine the role of other family members, especially fathers, in making vaccination decisions. Additionally, intervention studies aimed at both maternal health literacy and vaccine hesitancy could help develop more effective strategies to improve timely vaccination coverage. Further research could also explore multifactorial interventions that combine education, reminders, and culturally sensitive communication to improve adherence to vaccination schedules.

## Conclusion

The findings suggest that multiple factors influence mothers’ decisions regarding timely child vaccination. To address these complexities, targeted interventions such as mobile health reminders, community-based education programs, and integration of vaccination services into routine maternal health care are recommended. Since most mothers obtain information from healthcare providers, strengthening the role of health institutions in facilitating regular immunization visits is crucial. Moreover, fostering interdisciplinary collaboration among healthcare workers, educators, and media experts can help counter misinformation and build public trust. The observation that many mothers possess only moderate knowledge highlights the need to improve not only health knowledge but also the practical skills required to navigate health information and health systems effectively. By addressing both cognitive and structural barriers, these strategies may reduce vaccine hesitancy, prevent delays, and ultimately enhance child immunization coverage.

## Data Availability

Data will be provided by the corresponding author upon reasonable.
